# Challenges and Strategies Adopted for Remote Teaching of Biochemistry During the COVID-19 Pandemic: Protocol for a Scoping Review

**DOI:** 10.2196/59552

**Published:** 2025-01-31

**Authors:** Tatiane Iembo, Helena Landim Gonçalves Cristóvão, Emerson Roberto dos Santos, André Bavaresco Gonçalves Cristóvão, Nathália Bavaresco Gonçalves Cristóvão, Cíntia Canato Martins, Natália Almeida de Arnaldo Silva Rodrigues Castro, Fernando Nestor Facio Júnior, Antônio Hélio Oliani, Alba Regina de Abreu Lima, Vânia Maria Sabadoto Brienze, Doroteia Rossi Silva Souza, Júlio César André

**Affiliations:** 1 Laboratório Morfofuncional Faculdade de Medicina em São José do Rio Preto (FACERES) São José do Rio Preto Brazil; 2 Departamento de Biologia Molecular Faculdade de Medicina de São José do Rio Preto (FAMERP) São José do Rio Preto Brazil; 3 Departamento de Clínica Médica Universidade de Santo Amaro (UNISA) Santo Amaro Brazil; 4 Universidade Paulista (UNIP) São José do Rio Preto Brazil

**Keywords:** COVID-19, biochemistry, education, distance, teaching, educational technology, review, digital learning, virtual education, teaching tools, remote learning, social support, distance learning, remote teaching

## Abstract

**Background:**

In March 2020, the global landscape witnessed widespread upheavals in both socioeconomic and educational spheres due to the onset of the COVID-19 pandemic. With measures imposed to control the virus’s spread, educational institutions around the world embraced digital learning, introducing challenges in the adaptation to virtual education. This shift proved especially daunting in resource-limited nations with limited digital infrastructure.

**Objective:**

This scoping review aims to explore the experiences of biochemistry educators during the COVID-19 pandemic, focusing on successful pedagogical strategies used to overcome challenges in remote teaching. The goal is to compile valuable information applicable to health-related undergraduate and postgraduate courses.

**Methods:**

This review considers studies and experiences related to the transition to remote biochemistry education during the pandemic. It encompasses a variety of pedagogical approaches, including online teaching tools, interactive methods, and alternatives to practical laboratory classes. The search spans databases such as MEDLINE, the Cochrane Database of Systematic Reviews, and Joanna Briggs Institute (JBI) Evidence Synthesis, with a focus on identifying systematic or scoping reviews; however, none were identified in the preliminary search.

**Results:**

Starting in February 2022, the scoping review protocol was scheduled for completion by July 2024. From an initial pool of 1171 results, 85 articles were selected, with duplicate verification pending for the subsequent phase of the project. The findings from this review on biochemistry teaching strategies will be communicated using a combination of descriptive narrative, graphical, and tabular formats, emphasizing diverse pedagogical approaches pertinent to the subject. Dissemination will occur through regional and national scientific conference presentations, alongside publication in a peer-reviewed journal.

**Conclusions:**

This review aims to generate innovative pedagogical approaches and pinpoint learning activities, materials, and tools that support social and collaborative learning across various subjects, including biochemistry. Moreover, it will offer perspectives from students and educators on the implemented activities, with the intention of integrating them as supplementary methods to boost student participation, and thereby, improve learning outcomes and skill development.

**Trial Registration:**

Open Science Framework VZSA7; https://osf.io/VZSA7/

**International Registered Report Identifier (IRRID):**

DERR1-10.2196/59552

## Introduction

The COVID-19 pandemic, declared by the World Health Organization (WHO) in March 2020, caused significant impacts on both socioeconomic and educational fronts, disrupting the routine of in-person education worldwide [1]. Due to restrictions on people’s movement to prevent the spread of the coronavirus, schools and universities shifted to digital learning, which helped prevent 2% to 4% of COVID-19 deaths [2]. Consequently, educators and students were compelled to use digital platforms as universities faced the dilemma of either suspending activities indefinitely or restructuring through online education, with the latter prevailing [3,4]. This transition from traditional to virtual education posed challenges, requiring students and educators to adapt to digital platforms and teachers to develop some level of computer proficiency. In resource-limited countries, the limited digital infrastructure hindered online access, rendering online education less effective [3].

While online teaching tools were already used by various institutions, the widespread shift to this mode of education was abrupt for educators across all levels, from basic to postgraduate courses [4,5]. Therefore, pedagogical strategies had to be rapidly developed to achieve desired competencies through virtual learning without significant learning setbacks [4]. In addition, the need to identify optimal communication tools arose early on to facilitate practical aspects, such as ease of use, compatibility with teachers’ and students’ computer systems, and avoidance of additional costs for students [5]. This led to the adoption of online meeting programs, such as Microsoft Teams, Cisco Webex, GoToMeeting, Jitsi, and Zoom, on all available electronic devices and required changes in the education system to facilitate the development of students’ learning habits and critical thinking; this demanded quick adaptability and creativity from teachers in transitioning from a structured teaching method to an innovative one, especially in medical education [5]. Conventional methods of distance learning often involve prerecorded video lectures and theoretical materials, which may be insufficient to engage students and promote learning [6]. Furthermore, the unavailability of teachers to clarify doubts during and after the class may impede communication and content comprehension, a limitation recognized for several years and accentuated by the COVID-19 pandemic [6,7]. As an alternative to maintaining quality education and direct teacher-student contact, interactive pedagogical tools were used to promote active learning through student participation in online classes [8].

The rapid shift to distance learning during the pandemic presented significant global challenges, as evidenced by Lassoued et al [9], which identified pedagogical, technical, and financial obstacles affecting the quality of education in Arab universities, and by Jalali et al [10], who highlighted similar issues in Iran, where prosthetics and orthotics students faced unreliable internet and a lack of digital competence among both students and faculty, severely impacting the quality of online education. Similarly, Anwar et al [11] in Pakistan and Baticulon et al [12] in the Philippines identified several barriers to effective online learning, including low digital literacy, lack of institutional support, financial difficulties, unreliable internet access, the need for students to manage household responsibilities, and mental health challenges exacerbated by the pandemic, all of which hindered students’ ability to engage effectively with online education.

In the field of biochemistry education, specific examples of innovative approaches include the work of Botasini et al [13], who assessed the learning objectives of each practical biochemistry class at the University of Montevideo, distinguishing between those developing skills suitable for remote learning and those primarily requiring hands-on laboratory skills. Gasparello et al [14] replaced applied biochemistry practical classes with a remote activity simulating the use of different techniques for diagnosing SARS-CoV-2, the infectious agent responsible for the COVID-19 pandemic. They suggested continuing this strategy in person, with small groups of students to maintain social distancing. Similarly, Vasiliadou [15] proposed adopting virtual laboratories where practical classes were simulated, allowing students to perform them at their own pace, familiarizing them with health and safety regulations, and providing instant feedback. These virtual experiments could be conducted in groups, promoting social interaction and collaboration among students, crucial in a socially distant situation to facilitate communication and reduce feelings of isolation and loneliness.

Singh and Arya [1] reported the development of new approaches tested on various online platforms with a class of 200 biochemistry students. After using 4 different teaching strategies and analyzing student feedback, they combined these approaches with the flipped classroom method. This involved providing slides, quizzes, and support material links at least 24 hours before delivering a video lecture on a carbohydrate metabolism topic. Students’ questions were available to tutors before the online class, and the interactive phase of this hybrid strategy was recorded for students with poor connectivity to answer a related questionnaire later.

Challenges continued to emerge as the need to fulfill educational objectives clashed with the physical distance of students [8,16]. Practical laboratory classes, integral to health professionals’ training, posed a particular challenge. Teachers in these disciplines had to address how to teach practical content and skills remotely, leading to the implementation of various alternatives based on available digital tools [16].

As some researchers shared their experiences, such as Thibaut and Schroeder’s [17] guide for creating short cases for virtual case-based learning, the value of virtual learning as a complementary tool for the future became evident. In this regard, this scoping review aims to explore different experiences of biochemistry educators during the COVID-19 pandemic, identifying successful pedagogical strategies that engaged students in overcoming challenges posed by this conceptual and abstract discipline. This information can be compiled and used by educators in health-related undergraduate and postgraduate courses. Understanding complex molecular structures, intricate metabolic pathways, and subtle chemical interactions provides profound knowledge of the molecular basis of organism function and, consequently, the development, diagnosis, and treatment of diseases. A preliminary search of MEDLINE, the Cochrane Database of Systematic Reviews, and Joanna Briggs Institute (JBI) Evidence Synthesis did not identify any current or ongoing systematic reviews or scoping reviews on the topic.

## Methods

### Overview

The research methodology will involve the utilization of the Arksey and O’Malley [18] approach for scoping reviews, comprising the following steps: (1) identify the research question; (2) identify relevant studies; (3) perform study selection; (4) extract and chart the data; and (5) collate, summarize, and report the results. The scoping review methodology will be guided by this framework, and the protocol will follow the relevant PRISMA-ScR (Preferred Reporting Items for Systematic Reviews and Meta-Analyses Extension for Scoping Reviews) guidelines [19]. The review protocol was registered in the Open Science Framework [20].

### Identify the Research Question

As per Levac et al [21], the research question intended for exploration in this review has been clearly articulated and focused. The question being investigated in this exploratory literature review is “What were the challenges and strategies adopted for remote biochemistry teaching during the COVID-19 pandemic?”

### Participants, Concept, and Context Framework for Eligibility Criteria

The inclusion of eligible studies in this scoping review will be guided by the Participants, Concept, and Context framework [22].

#### Participants

This scoping review will include studies that involve students who participated in biochemistry courses at undergraduate or postgraduate levels, delivered remotely.

#### Concept

The focus of the study is to analyze the challenges and strategies adopted for remote biochemistry teaching, covering both theoretical and practical approaches, during the COVID-19 pandemic. Studies discussing pedagogical methods, online teaching tools, adaptation of laboratory practices, and other approaches to maintaining the quality of distance education will be considered.

#### Context

Only studies conducted during the COVID-19 pandemic will be included, as the objective is to explore how biochemistry teaching was adapted and the challenges faced during this specific period. Studies published in any language will be considered.

#### Exclusion

Duplicate articles, editorials, reviews, conference proceedings, theses, dissertations, and monographs will be excluded.

### Identify Relevant Studies

The literature search will be performed from January 2020 to December 2023, during the time period when articles related to tools used for remote teaching during the COVID-19 pandemic were published, in collaboration with a specialized librarian, using an iterative approach. Following the formulation of the question, keywords will be identified (“Teaching”; “Biochemistry”; “Distance Education”; and “COVID-19”) that managed to capture articles related to the topic, namely “translational medical research” (Medical Subject Headings terms) and “knowledge translation.”

The following databases will be consulted: National Library of Medicine (PubMed), MEDLINE, Scopus, Directory of Open Access Scholarly Resources, Education Resources Information Center, Directory of Open Access Journals, and Google Scholar, through descriptors and their synonyms, according to the Health Sciences Descriptors and Medical Subject Headings, for each item of the strategy (Tables 1 and 2). For the combination of descriptors, the Boolean terms AND, OR, and NOT will be considered.

**Table 1:** Search keywords and key terms used for this study.

Subjects	Subject and synonyms in Portuguese (DeCS^a^)	Subject and synonyms in English (MeSH^b^)
1	“Ensino” OR “Atividade de Treinamento” OR “Atividades Formativas” OR “Atividades de Capacitação” OR “Atividades de Formação” OR “Atividades de Treinamento” OR “Atividades de Treino” OR “Capacitação Acadêmica” OR “Didática” OR “Docência” OR “Formação Acadêmica” OR “Método de Ensino” OR “Métodos Pedagógicos” OR “Métodos de Ensino” OR “Pedagogia” OR “Treinamento Acadêmica” OR “Treino Acadêmico” OR “Técnica de Treinamento” OR “Técnicas Educacionais” OR “Técnicas Educativas” OR “Técnicas de Ensino” OR “Técnicas de Formação” OR “Técnicas de Treinamento” OR “Técnicas de Treino”	“Teaching” OR “Training Technique” OR “Training Technique” OR “Technique, Training” OR “Techniques, Training” OR “Training Technic” OR “Technic, Training” OR “Technics, Training” OR “Training Technic” OR “Pedagogy” “Pedagogies” OR “Teaching Method” OR “Teaching Method” OR “Method, Teaching” OR “Methods, Teaching” OR “Academic Training” OR “Training, Academic” OR “Training Activities” OR “Training Activity” OR “Activities, Training” OR “Activity, Training” OR “Techniques, Educational” OR “Educational Techniques” OR ‘Educational Technique” OR “Technique, Educational” OR “Educational Technics” OR “Educational Technic” OR “Technic, Educational” OR “Technics, Educational”
2	“Bioquímica”	“Biochemistry”
3	“Educação a Distancia” OR “Aprendizado Online” OR “Aprendizado a Distância” OR “Aprendizagem Online” OR “Aprendizagem a Distância” OR “Ciberaprendizagem” OR “Cursos por Correspondência” OR “Educação Online” OR “Ensino a Distância” OR “Formação à Distância” OR “Formação à Distância através das TIC” OR “Formação à Distância através das Tecnologias da Informação e das Comunicações” OR “Tele-Educação” OR “Tele-Educação Interativa” OR “Teleducação” OR “Teleducação Interativa” OR “Teleformação” OR “eLearning”	“Education, Distance” OR “Distance Education” OR “Distance Learning” OR “Learning, Distance” OR “Online Learning” OR “Learning, Online” OR “Online Education” OR “Education, Online” OR “Online Educations” OR “Correspondence Courses” OR “Correspondence Course” OR “Course, Correspondence”
4	“Covid-19” OR “COVID19” OR “Doença Viral COVID-19” OR “Doença pelo Novo Coronavírus (2019-nCoV)” OR “Doença por 2019-nCoV” OR “Doença por Coronavírus 2019” OR “Doença por Coronavírus 2019-nCoV” OR “Doença por Coronavírus-19” OR “Doença por Novo Coronavírus (2019-nCoV)” OR “Doença por Novo Coronavírus de 2019” OR“ Doença por Vírus COVID-19” OR “Epidemia de Pneumonia por Coronavírus de Wuhan” OR “Epidemia de Pneumonia por Coronavírus de Wuhan de 2019-2020” OR “Epidemia de Pneumonia por Coronavírus em Wuhan” OR “Epidemia de Pneumonia por Coronavírus em Wuhan de 2019-2020” OR “Epidemia de Pneumonia por Novo Coronavírus de 2019-2020” OR “Epidemia pelo Coronavírus de Wuhan” OR “Epidemia pelo Coronavírus em Wuhan” OR “Epidemia pelo Novo Coronavírus (2019-nCoV)” OR “Epidemia pelo Novo Coronavírus 2019” OR “Epidemia por 2019-nCoV” OR “Epidemia por Coronavírus de Wuhan” OR “Epidemia por Coronavírus em Wuhan” OR “Epidemia por Novo Coronavírus (2019-nCoV)” OR “Epidemia por Novo Coronavírus 2019” OR “Febre de Pneumonia por Coronavírus de Wuhan” OR “Infecção Viral COVID-19” OR “Infecção pelo Coronavírus 2019-nCoV” OR “Infecção pelo Coronavírus de Wuhan” OR “Infecção pelo SARS-CoV-2” OR “Infecção por 2019-nCoV” OR “Infecção por Coronavírus 2019-nCoV” OR “Infecção por Coronavírus de Wuhan” OR “Infecção por Novo Coronavírus de 2019” OR “Infecção por SARS Coronavirus 2” OR “Infecção por SARS-CoV-2” OR “Infecção por Vírus COVID-19” OR “Infecções por SARS-CoV-2” OR “Pandemia COVID-19” OR “Pandemia por COVID-19” OR “Pandemias por COVID-19” OR “Pneumonia do Mercado de Frutos do Mar de Wuhan” OR “Pneumonia por Coronavírus de Wuhan” OR “Pneumonia por Novo Coronavírus de 2019-2020” OR “Surto de Coronavírus de Wuhan” OR “Surto de Pneumonia da China 2019-2020” OR “Surto de Pneumonia na China 2019-2020” OR “Surto pelo Coronavírus 2019-nCoV” OR “Surto pelo Coronavírus de Wuhan” OR “Surto pelo Coronavírus de Wuhan de 2019-2020” OR “Surto pelo Novo Coronavírus (2019-nCoV)” OR “Surto pelo Novo Coronavírus 2019” OR “Surto por 2019-nCoV” OR “Surto por Coronavírus 2019-nCoV” OR “Surto por Coronavírus de Wuhan” OR “Surto por Coronavírus de Wuhan de 2019-2020” OR “Surto por Novo Coronavírus (2019-nCoV)” OR “Surto por Novo Coronavírus 2019” OR “Virose COVID-19” OR “covid-19”	“COVID19” OR “COVID 19” OR “SARS-CoV-2 Infection” OR “Infection, SARS-CoV-2” OR “SARS CoV-2 Infection” OR “SARS-CoV-2 Infections” OR “2019 Novel Coronavirus Disease” OR “2019 Novel Coronavirus Infection” OR “2019-nCoV Disease” OR “2019-nCoV Disease” OR “2019-nCoV Diseases” OR “Disease, 2019-nCoV” OR “COVID-19 Virus Infection” OR “COVID 19 Virus Infection” OR “COVID-19 Virus Infections” OR “Infection, COVID-19 Virus” OR “Virus Infection, COVID-19” OR “Coronavirus Disease 2019” OR “Disease 2019, Coronavirus” “Coronavirus Disease-19” OR “Coronavirus Disease 19” OR “Severe Acute Respiratory Syndrome Coronavirus 2 Infection” OR “SARS Coronavirus 2 Infection” OR “COVID-19 Virus Disease” OR “COVID 19 Virus Disease” OR “COVID-19 Virus Diseases” OR “Disease, COVID-19 Virus” OR “Virus Disease, COVID-19” OR “2019-nCoV Infection” OR “2019-nCoV Infection” OR “2019-nCoV Infections” OR “Infection, 2019-nCoV” OR “COVID19” “COVID-19 Pandemic” OR “COVID 19 Pandemic” OR “Pandemic, COVID-19” OR “COVID-19 Pandemics”

^a^DeCS: Health Science Descriptors.

^b^MeSH: Medical Subject Headings.

**Table 2:** Search strategy for databases used for this study.

Databases	Search details
National Library of Medicine (PubMed), MEDLINE, Scopus, Directory of Open Access Scholarly Resources, Education Resources Information Center, and Directory of Open Access Journal	(“Teaching” OR “Training Technique” OR “Training Technique” OR “Technique, Training” OR “Techniques, Training” OR “Training Technic” OR “Technic, Training” OR “Technics, Training” OR “Training Technic” OR “Pedagogy” “Pedagogies” OR “Teaching Method” OR “Teaching Method” OR “Method, Teaching” OR “Methods, Teaching” OR “Academic Training” OR “Training, Academic” OR “Training Activities” OR “Training Activity” OR “Activities, Training” OR “Activity, Training” OR “Techniques, Educational” OR “Educational Techniques” OR ‘Educational Technique” OR “Technique, Educational” OR “Educational Technics” OR “Educational Technic” OR “Technic, Educational” OR “Technics, Educational”) AND (“Biochemistry”) AND (“Education, Distance” OR “Distance Education” OR “Distance Learning” OR “Learning, Distance” OR “Online Learning” OR “Learning, Online” OR “Online Education” OR “Education, Online” OR “Online Educations” OR “Correspondence Courses” OR “Correspondence Course” OR “Course, Correspondence”) AND (“COVID19” OR “COVID 19” OR “SARS-CoV-2 Infection” OR “Infection, SARS-CoV-2” OR “SARS CoV-2 Infection” OR “SARS-CoV-2 Infections” OR “2019 Novel Coronavirus Disease” OR “2019 Novel Coronavirus Infection” OR “2019-nCoV Disease” OR “2019-nCoV Disease” OR “2019-nCoV Diseases” OR “Disease, 2019-nCoV” OR “COVID-19 Virus Infection” OR “COVID 19 Virus Infection” OR “COVID-19 Virus Infections” OR “Infection, COVID-19 Virus” OR “Virus Infection, COVID-19” OR “Coronavirus Disease 2019” OR “Disease 2019, Coronavirus” “Coronavirus Disease-19” OR “Coronavirus Disease 19” OR “Severe Acute Respiratory Syndrome Coronavirus 2 Infection” OR “SARS Coronavirus 2 Infection” OR “COVID-19 Virus Disease” OR “COVID 19 Virus Disease” OR “COVID-19 Virus Diseases” OR “Disease, COVID-19 Virus” OR “Virus Disease, COVID-19” OR “2019-nCoV Infection” OR “2019-nCoV Infection” OR “2019-nCoV Infections” OR “Infection, 2019-nCoV” OR “COVID19” OR “COVID-19 Pandemic” OR “COVID 19 Pandemic” OR “Pandemic, COVID-19” OR “COVID-19 Pandemics”
Google Scholar (in Portuguese)	(“Ensino” OR “Atividade de Treinamento” OR “Atividades Formativas” OR “Atividades de Capacitação” OR “Atividades de Formação” OR “Atividades de Treinamento” OR “Atividades de Treino” OR “Capacitação Acadêmica” OR “Didática” OR “Docência” OR “Formação Acadêmica” OR “Método de Ensino” OR “Métodos Pedagógicos” OR “Métodos de Ensino” OR “Pedagogia” OR “Treinamento Acadêmica” OR “Treino Acadêmico” OR “Técnica de Treinamento” OR “Técnicas Educacionais” OR “Técnicas Educativas” OR “Técnicas de Ensino” OR “Técnicas de Formação” OR “Técnicas de Treinamento” OR “Técnicas de Treino”) AND (“Bioquímica”) AND (“Educação a Distancia” OR “Aprendizado Online” OR “Aprendizado a Distância” OR “Aprendizagem Online” OR “Aprendizagem a Distância” OR “Ciberaprendizagem” OR “Cursos por Correspondência” OR “Educação Online” OR “Ensino a Distância” OR “Formação à Distância” OR “Formação à Distância através das TIC” OR “Formação à Distância através das Tecnologias da Informação e das Comunicações” OR “Tele-Educação” OR “Tele-Educação Interativa” OR “Teleducação” OR “Teleducação Interativa” OR “Teleformação” OR “eLearning”) AND (“Covid-19” OR “COVID19” OR “Doença Viral COVID-19” OR “Doença pelo Novo Coronavírus (2019-nCoV)” OR “Doença por 2019-nCoV” OR “Doença por Coronavírus 2019” OR “Doença por Coronavírus 2019-nCoV” OR “Doença por Coronavírus-19” OR “Doença por Novo Coronavírus (2019-nCoV)” OR “Doença por Novo Coronavírus de 2019” OR “Doença por Vírus COVID-19” OR “Epidemia de Pneumonia por Coronavírus de Wuhan” OR “Epidemia de Pneumonia por Coronavírus de Wuhan de 2019-2020” OR “Epidemia de Pneumonia por Coronavírus em Wuhan” OR “Epidemia de Pneumonia por Coronavírus em Wuhan de 2019-2020” OR “Epidemia de Pneumonia por Novo Coronavírus de 2019-2020” OR “Epidemia pelo Coronavírus de Wuhan” OR “Epidemia pelo Coronavírus em Wuhan” OR “Epidemia pelo Novo Coronavírus (2019-nCoV)” OR “Epidemia pelo Novo Coronavírus 2019” OR “Epidemia por 2019-nCoV” OR “Epidemia por Coronavírus de Wuhan” OR “Epidemia por Coronavírus em Wuhan” OR “Epidemia por Novo Coronavírus (2019-nCoV)” OR “Epidemia por Novo Coronavírus 2019” OR “Febre de Pneumonia por Coronavírus de Wuhan” OR “Infecção Viral COVID-19” OR “Infecção pelo Coronavírus 2019-nCoV” OR “Infecção pelo Coronavírus de Wuhan” OR “Infecção pelo SARS-CoV-2” OR “Infecção por 2019-nCoV” OR “Infecção por Coronavírus 2019-nCoV” OR “Infecção por Coronavírus de Wuhan” OR “Infecção por Novo Coronavírus de 2019” OR “Infecção por SARS Coronavirus 2” OR “Infecção por SARS-CoV-2” OR “Infecção por Vírus COVID-19” OR “Infecções por SARS-CoV-2” OR “Pandemia COVID-19” OR “Pandemia por COVID-19” OR “Pandemias por COVID-19” OR “Pneumonia do Mercado de Frutos do Mar de Wuhan” OR “Pneumonia por Coronavírus de Wuhan” OR “Pneumonia por Novo Coronavírus de 2019-2020” OR “Surto de Coronavírus de Wuhan” OR “Surto de Pneumonia da China 2019-2020” OR “Surto de Pneumonia na China 2019-2020” OR “Surto pelo Coronavírus 2019-nCoV” OR “Surto pelo Coronavírus de Wuhan” OR “Surto pelo Coronavírus de Wuhan de 2019-2020” OR “Surto pelo Novo Coronavírus (2019-nCoV)” OR “Surto pelo Novo Coronavírus 2019” OR “Surto por 2019-nCoV” OR “Surto por Coronavírus 2019-nCoV” OR “Surto por Coronavírus de Wuhan” OR “Surto por Coronavírus de Wuhan de 2019-2020” OR “Surto por Novo Coronavírus (2019-nCoV)” OR “Surto por Novo Coronavírus 2019” OR “Virose COVID-19” OR “covid-19”)

After conducting the search, the following will be included: studies conducted in English and Portuguese languages, with quantitative and qualitative approaches; primary studies, systematic reviews, meta-analyses, and meta-syntheses; and books and guidelines published in indexed sources that address the established question.

Duplicate articles, editorials, reviews, conference proceedings, theses, dissertations, monographs, undergraduate final projects, opinion publications, consensuses, retractions, editorials, websites, and advertisements disseminated will be excluded.

### Perform Study Selection

The citations gathered will be transferred to Mendeley Reference Manager for Desktop (version 2.109.0; Elsevier Ltd.) after the search, and the duplicates will be manually removed. Following this, 2 reviewers (TI and JCA) will independently assess titles and abstracts for eligibility using the Participants, Concept, and Context framework. Full texts of potentially relevant articles meeting the eligibility criteria will be retrieved for the 2 reviewers to scrutinize and compare against the inclusion criteria. In case of discrepancies during any phase of article selection between TI and JCA, resolution will be sought through discussion or consultation with ARdAL, the third reviewer. The final scoping review will feature a descriptive account of the search and study selection process alongside a PRISMA-ScR flow diagram [15].

### Extract and Chart the Data

The data from all eligible studies will be extracted and processed through data charting. This process involves extracting information from the selected full-text articles. A Microsoft Excel table will be created for this purpose, encompassing sections, such as title, authors, year of publication, geographic area, study objectives, pedagogical approaches, collaborative learning activities, resources adopted, and summary of findings.

### Collate, Summarize, and Report the Results

The characteristics of each identified study will be summarized and described (eg, author name, study location, and year of publication), along with the pedagogical approach used in teaching biochemistry; the learning activities, content, and resources used; and any evaluation by students of the strategies used by their professors.

## Results

Initiated in February 2022, the protocol for this scoping review was anticipated to wrap up by July 2024. The research process yielded 1171 results, from which 85 articles were selected. Verification of duplicates has not yet been conducted, and this will be carried out in the next stage of the work.

The outcomes of this scoping review on teaching strategies for biochemistry will be conveyed through a blend of descriptive narrative, graphical, and tabular formats. The presentation will be structured to underscore the various pedagogical approaches relevant to the field. These findings will be shared through regional and national scientific conference presentations, as well as publication in a peer-reviewed journal.

## Discussion

### Expected Findings

During the COVID-19 pandemic, the teaching of undergraduate and graduate courses in the health care field faced significant challenges [9]. The interruption of in-person laboratory activities and the need for quick adaptation to online teaching were some of the main obstacles [11,12]. Furthermore, the lack of access to laboratories and the disruption of clinical practice affected the development of essential practical skills for students [4].

However, some advantages were identified with the strategies adopted by teachers and institutions during the pandemic [8,17]. For instance, the use of online resources such as prerecorded videos and animated slides allowed for greater efficiency in academic work [1,5]. In addition, engaging in vital scientific activities such as reading articles and preparing presentations, coupled with the flexibility provided by remote learning, which enabled greater student participation, contributed to the cognitive development and enhancement of communication and technological skills of the students [1,4,5].

Considering these observations, continuing remote teaching strategies after the pandemic can bring various benefits [8]. The learning flexibility offered by online classes allows students to access content conveniently and effectively, promoting greater inclusion and expanding access to education [5,16]. Moreover, the possibility of continuous material review facilitates understanding and reinforces learning [1].

It should be emphasized that the use of educational technologies helps students develop essential digital skills for the current and future job market [5]. Therefore, blending educational strategies developed during the pandemic with in-person classes could bring additional opportunities for the improvement of teaching and learning not only in biochemistry but also in other disciplines [6,7].

In addition, this scoping review presents limitations. Specifically, there will be no evaluation of the quality of the studies included. There is a possibility of overlooking pertinent data, given that our search is restricted to published studies exclusively. We have chosen multiple sources and formulated meticulous search strategies to enhance the identification of eligible studies.

### Conclusions

The results of this review are expected to bring forth ideas for new pedagogical approaches and identify learning activities, content, and resources that facilitate social and collaborative learning processes, both for biochemistry and other disciplines. In addition, insights from students and teachers regarding the activities developed will be provided, aiming to apply them as additional strategies to enhance student engagement, and consequently, learning and the development of various skills.

## Abbreviations

JBI: Joanna Briggs Institute
PRISMA-ScR: Preferred Reporting Items for Systematic Reviews and Meta-Analyses Extension for Scoping Reviews
WHO: World Health Organization
